# Infection of highly insecticide-resistant malaria vector *Anopheles coluzzii* with entomopathogenic bacteria *Chromobacterium violaceum* reduces its survival, blood feeding propensity and fecundity

**DOI:** 10.1186/s12936-020-03420-4

**Published:** 2020-10-02

**Authors:** Edounou Jacques Gnambani, Etienne Bilgo, Adama Sanou, Roch K. Dabiré, Abdoulaye Diabaté

**Affiliations:** 1 Institut de Recherche en Sciences de La Santé (IRSS) / Centre Muraz, Bobo Dioulasso, Burkina Faso; 2 Université Nazi Boni / Centre Muraz, Bobo-Dioulasso, Burkina Faso

**Keywords:** *Chromobacterium violaceum*, *Anopheles coluzzii*, Blood feeding, Fecundity, Malaria, Burkina faso

## Abstract

**Background:**

This is now a concern that malaria eradication will not be achieved without the introduction of novel control tools. Microbiological control might be able to make a greater contribution to vector control in the future. The interactions between bacteria and mosquito make mosquito microbiota really promising from a disease control perspective. Here, the impact of *Chromobacterium violaceum* infections, isolated from both larvae and adult of wild-caught *Anopheles gambiae *sensu lato mosquitoes in Burkina Faso, was evaluated on mosquito survival, blood feeding and fecundity.

**Methods:**

To assess entomopathogenic effects of *C. violaceum* infection on mosquitoes, three different types of bioassays were performed in laboratory. These bioassays aimed to evaluate the impact of *C. violaceum* infection on mosquito survival, blood feeding and fecundity, respectively. During bioassays mosquitoes were infected through the well-established system of cotton ball soaked with 6% glucose containing *C. violaceum*.

**Results:**

*Chromobacterium violaceum* kills pyrethroid resistant *Anopheles coluzzii* (LT80 of 8.78 days ± 0.18 at 10^8^ bacteria cell/ml of sugar meal). Interestingly, this bacterium had other negative effects on mosquito lifespan by significantly reducing (~ 59%, P < 0.001) the mosquito feeding willingness from day 4-post infection (~ 81% would seek a host to blood feed) to 9- day post infection (22 ± 4.62% would seek a host to blood feed). Moreover, *C. violaceum* considerably jeopardized the egg laying (~ 16 eggs laid/mosquito with *C. violaceum* infected mosquitoes *vs* ~ 129 eggs laid/mosquito with control mosquitoes) and hatching of mosquitoes (a reduction of ~ 22% of hatching rate with *C. violaceum* infected mosquitoes). Compared to the bacterial uninfected mosquitoes, mosquitoes infected with *C. violaceum* showed significantly higher retention rates of immature eggs and follicles.

**Conclusion:**

These data showed important properties of Burkina Faso *C. violaceum* strains, which are highly virulent against insecticide-resistant *An. coluzzii*, and reduce both mosquito blood feeding and fecundity propensities. However, additional studies as the sequencing of *C. violaceum* genome and the potential toxins secreted will provide useful information render it a potential candidate for the biological control strategies of malaria and other disease vectors.

## Background

In the last three years, many countries have reported significant increases in malaria cases, according to the World Health Organization (WHO) latest malaria report [1]. In 2018, the number of malaria cases worldwide was 228 million cases [2], which is a reduction from 237 millions in 2010, but since then progress towards global elimination of malaria has been stalling [3, 4], with 93% of cases occurring in the WHO African region [2]. The reasons for the slowdown differed across specific regions and countries, but contributing factors included insufficient funding, a lack of interventions to prevent spread of the disease, risks posed by conflict in malaria endemic regions, irregular climate patterns [4], and the rapid emergence of both parasite and mosquito vector resistance to drugs and insecticides [1]. Emergence and spread of resistance to pyrethroids, organophosphates and carbamates is a particular threat, as most malaria control programmes rely heavily on these broad-spectrum insecticides to reduce vector populations [5, 6].

As a consequence of these growing problems, the WHO called for research and development of alternative approaches in controlling vector-borne diseases, thus decreasing the usage of insecticides [7]. Integrated Vector Management (IVM) efforts are now oriented towards controlling *Anopheles* either at the larval stage and/or at the adult stage by means of microbial control, namely fungi and bacteria [8, 9]. Many of these approaches are now focusing on the use of genetically engineered microorganisms to either block the development of the malaria parasite within the *Anopheles* vector [10–14], or target the vector itself [10, 15]. Despite intensive efforts to develop entomopathogenic bacteria as biocontrol agents against malaria vectors, the strains under investigation have not met expectations due to some functional and practical limitations [8, 9]. For example, bacteria such as *Bacillus thuringiensis israelensis* (Bti) and *Bacillus sphaericus* (Bs) show no residual persistence post-application [8]. Among promising entomopathogenic bacteria *Wolbachia*, only a few strains are known to be associated with *Anopheles gambiae* [16].

Interestingly, Ramirez and collaborators in 2014, showed that *Chromobacterium* sp. Panama (*C.* sp_P) isolated from the midgut of field-collected *Aedes aegypti*, has unique properties: it can kill larvae and adults of multiple mosquito species, and it exerts in vitro anti-*Plasmodium* and anti-dengue virus activity suggesting that it could be a highly potent candidate for developing tools against current and future mosquito-borne diseases [10]. *Chromobacterium violaceum* is a Gram-negative facultative anaerobic and non sporing-coccobacillus bacterium. This bacterium is rarely human pathogenic*.* It is part of the normal flora of water and soil of tropical and sub-tropical regions of the world. It produces a natural antibiotic called violacein [17]. Some strains of *Chromobacterium* have already been developed for agricultural pest control [18]. Short et al*.* have shown that *Chromobacterium* sp. Panama *C*.sp_P exposure have important effects on mosquito fitness and mosquito physiology including some transgenerational impacts [19]. In the same study, they have also shown that mosquito exposure to cell-free *C.*sp_P-conditioned media could elicit detoxification, xenobiotic response, and stress response genes within female mosquito midgut [19]. This phenomenon is also shown when mosquitoes are exposed to common chemical insecticides used for vector control and this highlights the potential of using *Chromobacterium* for vector borne diseases control, including malaria.

In this study, the impact of infection of malaria vector (*An. coluzzii*) with an indigenous burkinabè strain of *C. violaceum* isolated from both wild caught adults and larvae of *An. gambiae* was examined. Using a logistically simple method of infection, cotton balls soaked with sugar meal containing bacteria, assessments of the pathogenicity of this local strain of *C. violaceum* against adult mosquitoes and its impact of mosquito blood feeding and fecundity were carried out.

## Methods

### Bacteria strain

The *Chromobacterium violaceum* strain used for bioassays is isolated from both field collected larvae and cuticles of adult mosquitoes (*An. gambiae s.l.*) from Bana (11° 9′ 41"N, 4° 10′ 30" W), Soumousso (11°04′ N, 4°03′ W) in western Burkina Faso. Homogenates from dead mosquitoes were firstly plated out onto chocolate + polyvitek agar and bromocresol purple agar. Then, 24 and 48 h, bacterial species were isolates and species of *C. violaceum* were identified using the VITEK2 system (Additional file 1) in the laboratory at Centre Muraz.

### Mosquito colonies and PCR determination of *kdr* levels

For bioassays, F1 progeny of *An. coluzzii* reared from larval collections at Kou Valley (11°23′ N, 4°24′ W) was used. Mosquitoes from these areas are highly resistant to multiple insecticides [20]. Only non-blood-fed females, 2–5 days old, were used for bioassays. All bioassays were carried out at 25 ± 2 °C and 80 ± 10% relative humidity.

The *kdr* gene prevalence within a subsample of mosquitoes (180 mosquitoes) was performed using the PCR protocol and primer sequences previously described [21]. The mutation L1014F was only analysed because it is the commonest in West Africa, whereas the L1014S mutation is confined to East Africa [21]. The primers AgD1 (5′-ATA GAT TCC CCG ACC ATG-3′) and AgD3 (5′-AAT TTG CAT TAC TTA CGA CA-3′) amplified the resistant allele yielding 195 bp fragments. The susceptible allele was assayed using primers AgD2 (5′-AGA CAA GGA TGA TGA ACC-3′) and AgD4 (5′-CTG TAG TGA TAG GAA ATT TA-3′), which amplified a 137 bp fragment. The primer set AgD1 and AgD2 amplified a ubiquitous 293 bp fragment as a positive control. During amplification, denaturation was set at 94 °C for 3 min followed by 35 cycles of denaturation, annealing and elongation (94 °C for 30 s, 55 °C for 30 s, 72 °C for 10 s, respectively). The final elongation was set at 72 °C for 5 min.

### Bacterial infection formulation

**Fig. 1:**
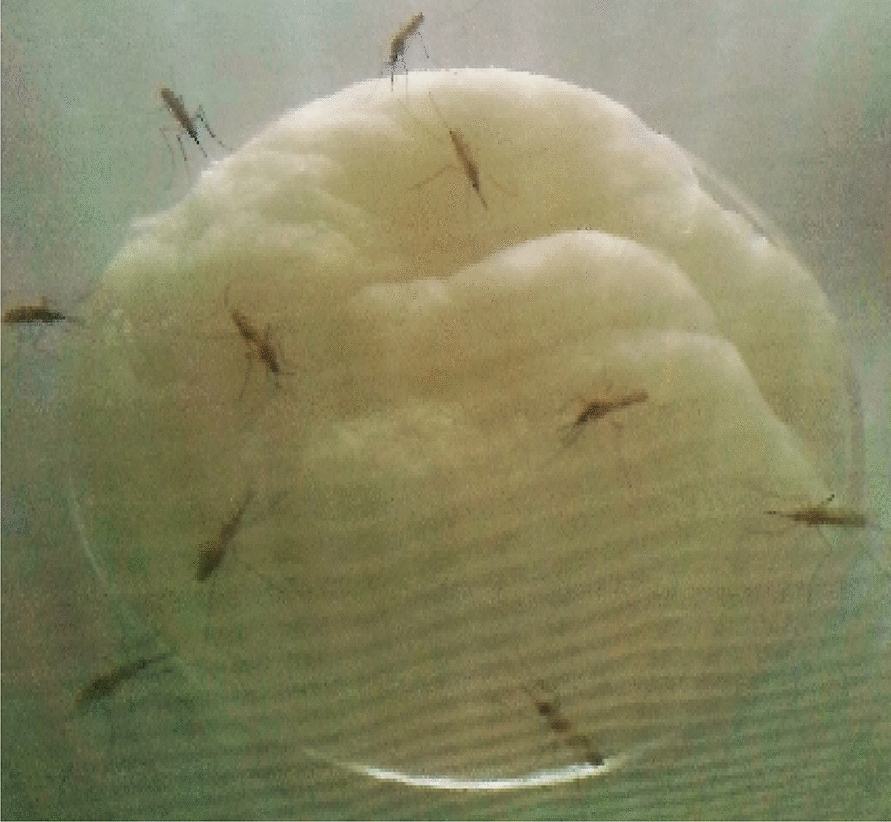
*Anopheles coluzzii* mosquitoes feeding upon a cotton ball soaked with 6% glucose containing *Chromobacterium violaceum*

Mosquitoes used for bioassays were not treated with antibiotics. They were maintained on 6% glucose for 2–5 days post emergence. Mosquitoes were then starved overnight and fed for 24 h on cotton balls moistened with a 6% glucose solution containing *C. violaceum* at desired concentrations (bacterial cells /ml) regarding the purposes of the bioassays (Fig. 1). The numbers of bacterial cells were determined by counting through improved Neubauer haemocytometre.

### Bioassays

#### Bioassay1: Evaluation of *C. violaceum* entomopathogenic activity upon mosquito ingestion

To assess the virulence of *C. violaceum* against adults *An. coluzzii*, 400 newly emerged females were exposed to feed upon cotton balls moistened with a 6% glucose solution containing, respectively, 10^5^,10^6^,10^7^ and 10^8^ bacterial cells/ml in a cage (15 × 15 × 15 cm) for 24 h. For each concentration, four replicates of 25 mosquitoes/replicate.

Four replicates of control batches of mosquitoes (25 mosquitoes/replicate) were exposed to blank cotton balls soaked with 6% glucose (without any bacterial cells). After 24 h of exposure to treated or untreated cotton balls, mosquitoes were transferred to other new cages (15 × 15 × 15 cm) and fed with sterile 6% glucose. Daily mortality was recorded over two weeks. Cadavers were immediately removed from their cages and each was washed once for 20 s with 1% sodium hypochlorite and twice with sterile distilled water for 40 s. Washed cadavers were individually crushed in 200 µl of sterilized phosphate saline buffer (PBS), homogenized. Hundred microlitre (100 µl) of each homogenate was then plated onto 2 different media (Chocolate + polyvitek agar and Bromocresol purple agar). Infection from *C.violaceum* was then confirmed 48 h after incubation and also using VITEK2 system.

#### Bioassay 2: Blood feeding choice tunnel to assess the impact of *C. violaceum* infection on mosquito host-seeking blood-feeding propensity

**Fig. 2:**
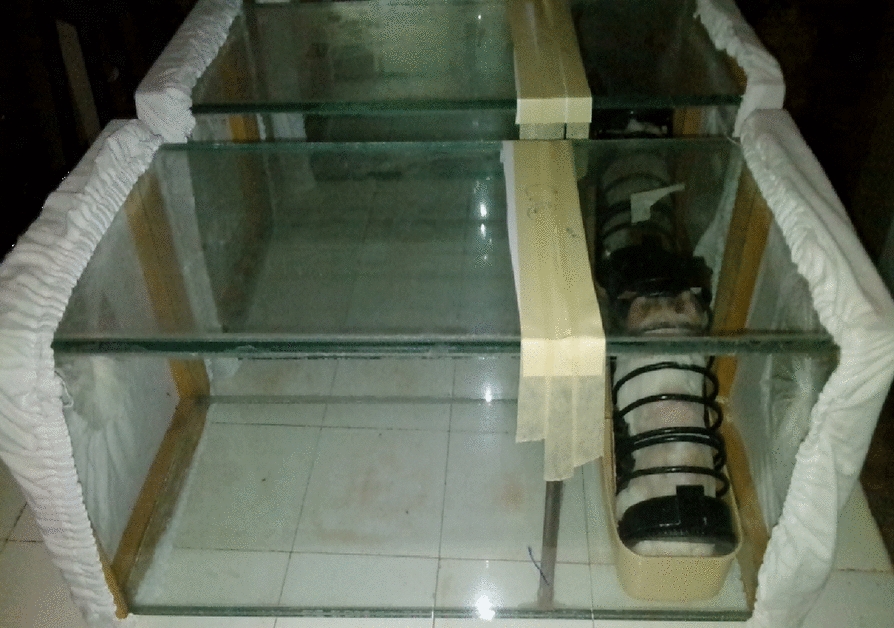
Design for experiments testing host-seeking behavior using guinea pigs and a tunnel choice chamber with nine small holes cut into a barrier between compartments

Fifty non-blood-fed infected or uninfected female mosquitoes (*An. coluzzii*) for each of the four replicates of the tunnel test were released into the tunnel to evaluate the impact of *C. violaceum* infection on mosquito host-seeking blood-feeding propensity. The bacterial dose (10^6^ bacterial cells / ml) was used for the tunnel bioassay. This dose is suboptimal for killing *An. coluzzii*, with resulting LT50 of 9 days post infection. Three to nine days post infection mosquitoes were used for the bioassays. The control mosquitoes were infected with blank distilled water solution without any bacteria. The protocol described by Bilgo et al*.* was followed [22] with some modifications. The tunnel is basically a 60-cm long; glass choice tunnel (25 × 25 cm area) was used for blood feeding assays (Fig. 2). A 25-cm square of polyester netting was fitted at one end of the tunnel as a compartment. A netted barrier was placed one-third along the length of the glass tunnel separating the tubes into short and long sections. The barrier was 400 cm^2^ (20 × 20 cm), with nine 1 cm diameter holes for passage; one hole was located at the centre of the square and the other eight were equidistantly located 5 cm from the border. This choice chamber is designed as a miniaturized proxy for a traditional West African home. The largest section of such a home is the veranda that serves as a sitting area. This corresponds to the first compartment of the tunnel (40 cm long). The second smaller part of a traditional house is the bedroom where residents sleep under bed netting corresponds to the smaller compartment of the tunnel (20 cm long). The guinea pig was placed within this compartment to represent a sleeping occupant at night (Fig. 2). Fifty non-blood-fed female mosquitoes (*An. coluzzii*) were released into the long section of the tunnel. In this design, female mosquitoes are normally attracted through the barrier into the smaller compartment by the guinea pig to blood feed. In full darkness between 6 pm and 6 am, mosquitoes interested in blood feeding were free to fly through the tunnel, locate the holes and pass through them to reach the guinea pig. The location of mosquitoes after this period was recorded, and those in the section closest to the guinea pig were considered to have interest in blood feeding. Mosquitoes were removed from each section of the tunnel and counted separately.

The tunnel bioassays were carried out at 27.34 °C average temperature (range 27.06–27.60 °C). The average relative humidity was 76.60% (range 75.90–77.00%). The mortality during the assay was recorded, but only live mosquitoes were considered for analysis.

#### Bioassay 3: Determination of the effect of *C. violaceum* infection on mosquito fecundity

An overall 75 three days old inseminated females mosquitoes were exposed to *C. violaceum* at 10^6^ bacterial cells/ml according to the infection procedure described above. Control batches of inseminated female mosquitoes were exposed to blank sugar meal without any bacterial cells. For control and treated mosquitoes, as field mosquitoes of this species do not mate enough in captivity, after adult emergence, inseminations using a force-mating technique between virgin males and females were done according the detail protocol described in MR4 2014 [23]. Control and treated female mosquitoes received then two blood meals, 48 and 72 h after insemination. As regard the blood feeding on rabbit, the abdomens of mosquitoes were observed to ensure that are they are filled with blood. After the two blood meals, each female was transferred in an individual small cup containing a blotting paper. A layer (~ 1 cm) of tap water has stayed on the roof of the paper in order to promote eggs laying and hatching. The number of eggs laid per mosquito was scored for the following seven day. The viable larvae of first instars from eggs were also counted 2–3 days after hatching.

### Ovary dissection

Experiments were therefore performed in order to look at the impact of *Chromobacterium violaceum* on the development of ovaries within both infected inseminated females and uninfected mosquitoes. After the blood meals as above, inseminated females were anesthetized onto cold (− 20 °C) for five to ten minutes. Then we dissected individually their ovaries under a microscope as described in detailed in MR4 [22] (75 mosquitoes overall). The aspects of the ovaries were examined under microscope at 40 times magnification. Leica software (LAS-EZ-V3-3-0 for PC) was used to take pictures of the aspects of ovaries.

### Data analysis

All data were entered into Microsoft Windows Excel 2013, checked for accuracy, then imported to R studio version 2.11.1 for data manipulation, visualization and statistical analysis (Additional file 2). Using Fisher’s exact test, P < 0.05 was accepted as statistically significant.

The main parameters were calculated at each time point as below:

$$Survival\, rate=(1-\frac{total\,dead\,mosquitoes}{ total\,mosquitoes} )x 100$$

$$Blood\,feeding\,rate=\frac{total\,blood\,fed\,mosquitoes }{total\,mosquitoes( blood\,fed+unfed)} x 100$$

$$Rate\,of\,egg\,laid/mosquito =\frac{Total\,eggs\,laid }{total\,females}$$

$$Eggs\,hatching rate=\frac{total\,egg\,hatches}{total\,egg\,laid} x 100$$

For these main parameters the standards errors (SE) for all replicates were calculated using the library plyr of R 3.2.4:

$$SE (length\,Parameter)=\frac{standard\,deviation(parameter)}{\surd (Length(parameter))}$$

LT_80_ survival for treatments and concentrations were determined using generalized linear model (GLM) approach.

Pairwise t. test comparisons with correction of ohm were used to compare the mean number of mosquitoes per treatments that blood fed, the number of eggs laid and the number of egg hatched respectively. For all bioassays, mosquitoes were considered alive if they could stand upright and dead if they were unresponsive to stimuli following the 2013 recommendations by the WHO Pesticides Evaluation Scheme [24].

### Ethics statement

All experiments with guinea pigs and rabbits were carried out in strict accordance with the recommendations in the Guide for the Care and Use of Laboratory Animals of the National Institutes of Health [25]. In addition, the protocols followed the IRSS Animal Welfare Assurance A5926-01. Trained personnel and veterinarians cared for animals involved in this study and all efforts were made to minimize suffering. All works with *C. violaceum* were performed under biosafety containment level II requirements.

## Results

### Entomopathogenic effect of *C. violaceum* on *An. coluzzii* survival

**Fig. 3:**
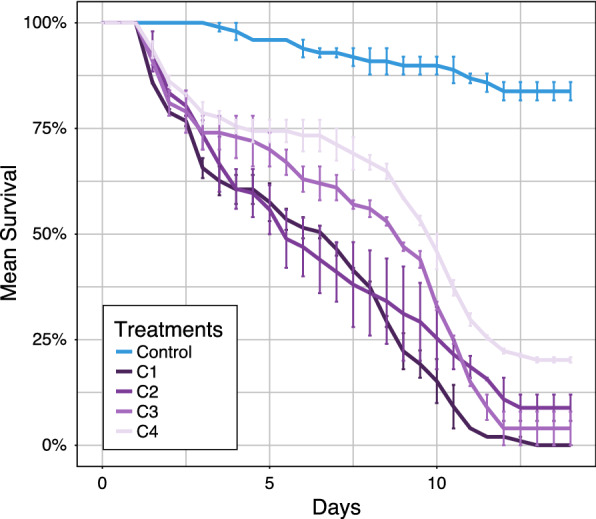
Survival curves of mosquitoes exposed to different concentrations of *C. violaceum*

Overall, 400 female mosquitoes of *An. coluzzii* were infected with serial dilution concentrations of *C. violaceum* from 10^8^ to10^5^ bacterial cells/ml. The level of *kdr* resistance within the subsample of first generation (F1) progeny of *An. coluzzii* that we used for bioassays was 98.3%. Within ~ 9 days post-infection, more than 80% of mosquitoes exposed to the higher concentration to 10^8^ bacterial cells/ml were dead, so significantly faster (P < 0.05) than those exposed to the 3 lower concentrations (Fig. 3, Table 1).

**Table 1:** LT_80_ survival values of mosquito treated with *C. violaceum*

Treatments	LT_80_ Mean (Days)	S.E. (Days)	Significance LT_80_^1^
Control	*–*	*–*	*–*
C1	8.78	0.12	a
C2	10.16	0.51	b
C3	10.85	0.42	b
C4	13.46	0.01	c

*SE* standard error of the mean

^1^Pairwise comparison of LT_80_ values per spraying conidia suspension concentrations: Treatments without letters in common are significant at p < 0.05. C1, C2, C3 and C4 are 1 × 10^8^ bacteria cells /mL, 1 × 10^7^ bacteria cells /mL, 1 × 10^6^ bacteria cells /mL and in 1 × 10^5^ bacteria cells /mL in 6% glucose meal, respectively. Control is exposed to blank cotton balls soaked 6% glucose meal (without any bacterial cells)

However there is no difference (P = 0.22) in term of virulence (LT_80_) between 10^7^ and 10^6^ bacterial cells/ml with LT80 value of 10.16 ± 0.51 and 10.85 ± 0.42 days respectively (Table 1). The lower concentration 10^6^ bacterial cells / ml did not reach the LT_80_ threshold out of 13 days. Observing the survival over 2 weeks, mosquitoes of uninfected control group never dropped below 84.2% (Fig. 3).

### Impact *of C. violaceum* infection on females *An. coluzzii* blood feeding propensity and malaria transmission interruption

**Fig. 4:**
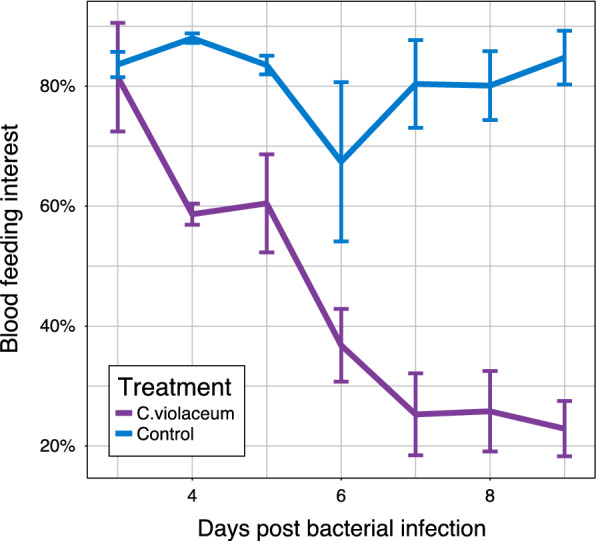
Impact of bacterial infection on blood feeding at 3–9 days post-infection with *C. violaceum*

Willingness to blood feed was also tested. Host-seeking (blood feeding) interest was quantified as the percentage of the mosquito population choosing to enter and bite the host (guinea pig). At three day post-infection, 83.6% (185/189) of untreated (controls) and 82.12% (147/179) treated (*C. violaceum*) mosquitoes flew toward the guinea pig and took their blood meal with no significant differences between treatments. The willingness of mosquitoes in the control group to blood feed did not change over the course of the experiment (from day 3 to day 9 post infection), (Fig. 4). In contrast, significantly (p < 0.05) fewer mosquitoes treated with *C.violaceum* flew and had the blood meal after from day 4-post infection to day 9-post infection as compared to Control (P < 0.05), (Fig. 4). From day 4 to 9 days post infection, an important reduction (59%) of blooding interest with mosquitoes treated with *C. violaceum* was observed. By nine-day post infection the number of *C. violaceum* infected mosquitoes proportion in the guinea pig choice chamber (22 ± 4.62%) was not significantly different than the 30% entering the chamber in the absence of a guinea pig.

**Fig. 5:**
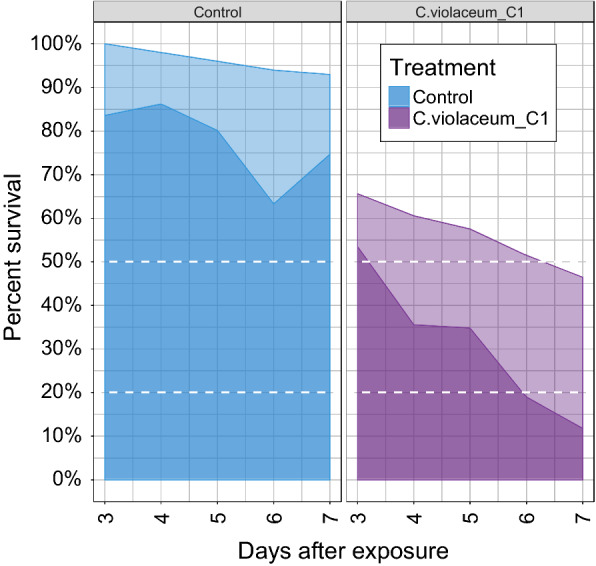
Mortality and transmission of mosquitoes exposed *to C. violaceum*

The results above suggest a pre-lethal advantage of using *C. violaceum* for mosquito control. With this information, the measured proportion of mosquitoes interested in blood feeding onto the mortality of mosquitoes was projected between 3 and 7 days post infection in order to identify the fraction capable of malaria transmission (Fig. 5). In contrast to untreated mosquitoes, by day 6, *C. violaceum* infected mosquitoes passed the threshold in both metrics (18.95% malaria transmission and > 80% mortality) for the 80% control threshold suggested by the WHO for a successful vector control agent.

### Impact of *C. violaceum* infection on females *An. coluzzii* fecundity

**Fig. 6:**
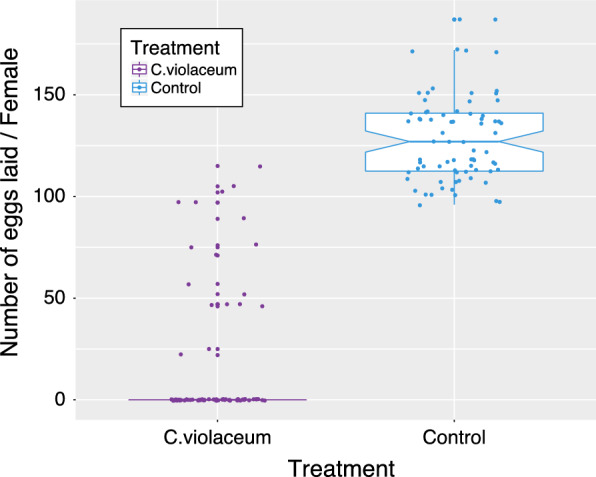
Impact of bacterial infection (*C. violaceum*) on mosquitoes (*An. coluzzii*) egg laying propensity

**Fig. 7:**
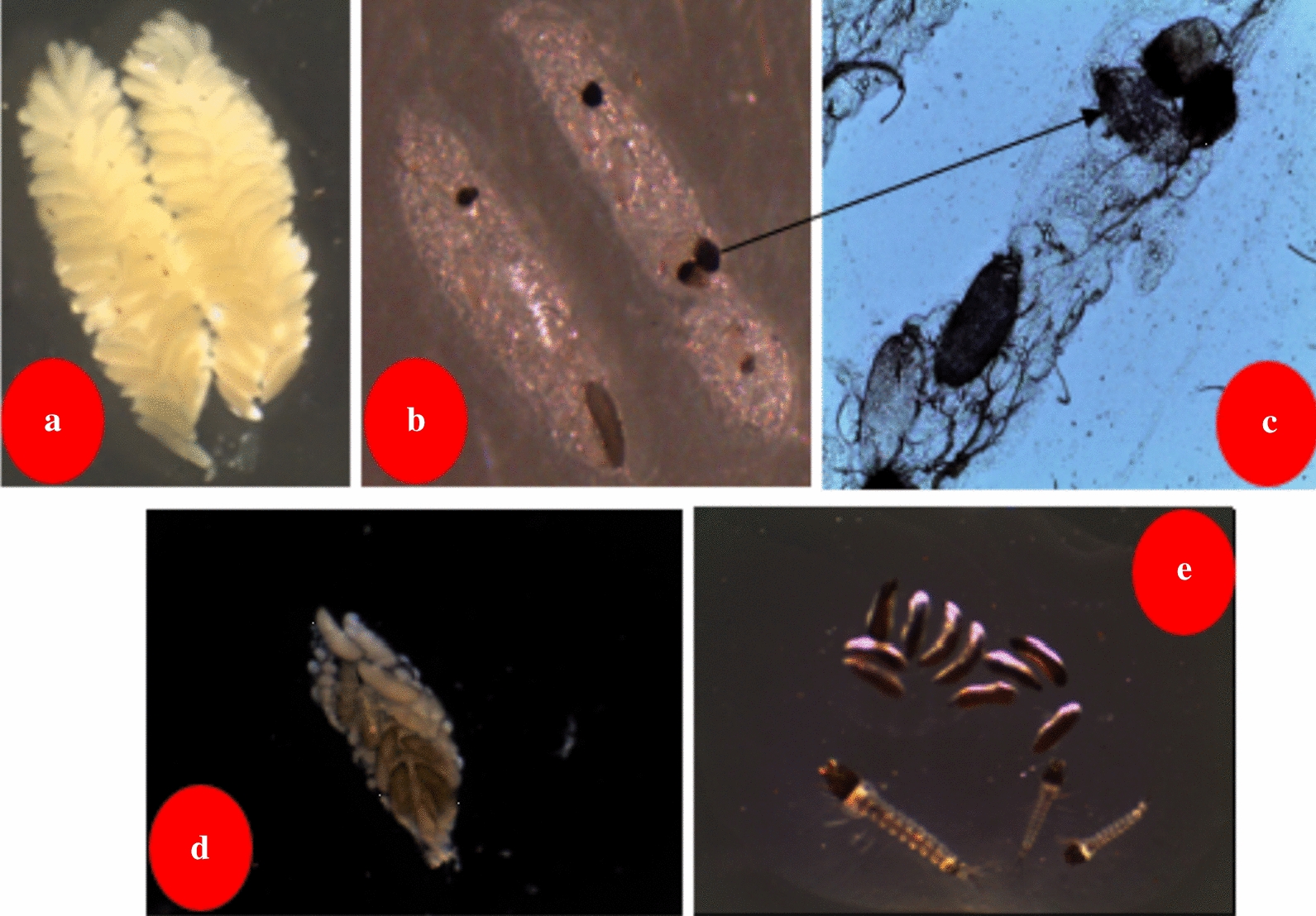
Impact of *C. violaceum* infections on ovarian follicles and fertilized egg maturations in *An. coluzzii* mosquitoes. Legend: Eggs of an uninfected female (**a**); Follicles and fertilized eggs of infected female with *C. violaceum* (**b**–**d**); non-viable eggs and larvae of an infected female (**e**)

**Fig. 8:**
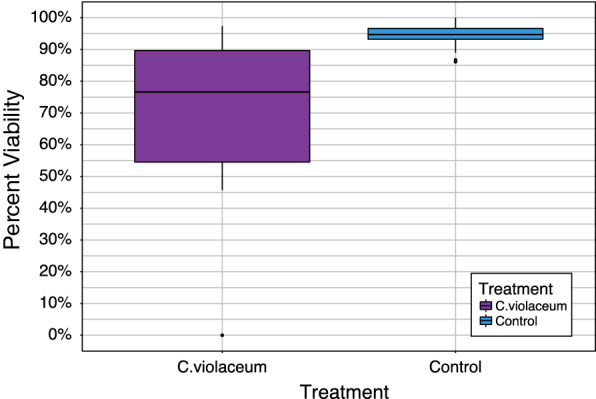
Effect of bacterial infection (*C. violaceum*) on larval vitality rates in mosquitoes (*An. coluzzii*)

Figure 6 shows the pattern of the number of eggs laid by 75 inseminated females infected with *C. violaceum* and 75 inseminated non-infected females (Control). Overall, 1,170 eggs were laid by 17 females in the *C. violaceum* treated group while 9,648 eggs were laid by 75 females in the control group. Between *C. violaceum* treated mosquitoes and uninfected mosquitoes, a significant difference in terms of mean egg laying propensity per female (P < 0.001) was observed. A microscopic examination of the aspects of ovaries of inseminated females infected with *C. violaceum* showed a colonization of ovaries of females by *C. violaceum* leading to eggs and ovarian deformities (Fig. 7). Regarding the hatching rate and viability of first instar larvae from the two groups, significant reduction of ~ 22% in the *C.violaceum* treated mosquitoes was also found (Fig. 8).

## Discussion

*Chromobacterium violaceum* has shown an oral toxicity in a population of malaria vector *An. coluzzii* that is highly resistant to pyrethroids. With a medium concentration, this local strain of *Chromobacterium* surpassed the mortality of 80% thresholds of WHO over a week. *Chromobacterium violaceum* exerted an important entomopathogenic activity even in the presence of other microorganisms from mosquito microbiota, because non-aseptic mosquitoes were used during bioassays. Indeed, The strain of Chromobacterium used in this study could be a natural and specific *Anopheles*-pathogens because it was originally isolated from wild larvae and adults of *An. gambiae s.l.* in western Burkina Faso. In contrast, previous mosquito-*Chromobacterium* strains were isolated from *Aedes aegypti* midgut [10]. The current strain of *Chromobacterium* virulence results corroborate those found by Ramirez et al*.* that also showed a low survival rate of *An. gambiae* and *Ae. aegypti* after a blood meal containing *Chromobacterium sp* at 10^8^ bacterial cells/ml. However the entomopathogenic effect of *Chromobacterium* species upon *An. gambiae s.l.* is still to be elucidated. A number of potential virulence factors may contribute to mosquitocidal effect, including production of the pigment violacein, siderophores, hydrogen cyanide, and secreted chitinases [26]. In addition, some strains *Chromobacterium* are capable of forming biofilms in vitro, though whether biofilm formation occurs within the mosquito midgut remains untested [10]. Bacterial biofilms are structured clusters of bacterial cells coated with a polymeric matrix and attached to a surface. The biofilm protects bacteria and allows them to survive in harsh environmental conditions and to resist the immune response of the host. The ability to form a biofilm is now recognized as a characteristic of many entomopathogenic microorganisms [27].

The results from the present study indicated that the rapid death of mosquitoes from *C. violaceum,* however is not the only part of the story. Once infected with *C. violaceum*, mosquitoes are less inclined to blood feed which mainly appear a common effect of fungal infection in mosquitoes and non for bacterial infections [23, 28]. The reduction of mosquito willingness to blood feed appears stronger as the bacterial infection progresses and can contribute to significant reductions in host feeding as early as day 6, essentially accelerating transmission blocking. What is interesting here, is that when the effects of blood feeding are added in risk of malaria transmission is essentially reduced to zero within 4 day of bacterial exposure and never recovers. *Chromobacterium violaceum* infection in mosquitoes could share the same physiological impact in term of blood feeding reduction as with fungi. Fungal infection actually increases mosquito metabolic rate and reduces flight propensity and flight stamina. Poor flight performance has been strongly associated with reductions in the mobile energy reserves of the host [29, 30]. Further specific studies are needed to access the impact of *C. violaceum* infection on mosquito flight ability associated with blood feeding propensity to withdraw more conclusions.

One of the important results this study is the disruption of mosquito reproduction after infection with *C. violaceum*. Indeed, bioassays showed that *C. violaceum* inhibited egg development in the ovaries of *An. coluzzii* mosquitoes and Short et al*.* [19] have shown that the exposure to *Chromobacterium* (*C.* sp_P) impacts female mosquito transgenerational fitness with no change in fecundity. From the current study, egg exposures to *C. violaceum* appear to negatively impact ovarian follicles development. Female mosquitoes require blood meals for their egg maturations. Newly emerged female mosquitoes come out from the larval stage with theirs follicles at first stage (Stage I) need blood meals to complete the development of their ovaries to the follicle stage V. In the current experiments, 24 h of exposure to *C. violaceum* reduced and subsequently stopped the maturation of the follicle because they have not reached the stage V for the most of them. *Chromobacterium violaceum* could secrete a hormone or substance that directly affects the development of the ovaries and eggs. This substance could indirectly inhibit the action of the juvenile hormones (JHs) in mosquitoes. The juvenile hormones are mainly responsible for the development of ovaries and eggs in mosquitoes [31]. The biochemical and physiological mechanisms governing the reduction of mosquito fecundity following *C. violaceum* infection remain to be elucidated. On other hand, a few *C.violaceum* infected females of *An. coluzzii* that were able to lay a couple of eggs were not viable or had a low hatching rate. *C. violaceum* infection seems to share these vertical transmission (mother to offspring) characteristics with some bacteria as *Serratia sp*, *Pantoa agglomerans*, *Asaia* sp [14, 31–35]. In addition, experiments are needed to be performed to check if *C. violaceum* plays a sort of cytoplasmic incompatibility properties as some strains of *Wolbachia* [36] within their mosquito host*.* In the simplest case, crossing-bioassays between *C. violaceum* infected and uninfected *An. coluzzii* in order to see if early embryonic arrest occurs when uninfected females mate with infected males.

## Conclusion

There is a global consensus that in the fight against malaria a ‘magic bullet’ does not exist. The disease can only be controlled by the coordinated deployment of as many weapons as possible. Here, we have isolated and identified a *C. violaceum* strain from *An. gambiae s.l.* which exerted an important entomopathogenic effect upon mosquito ingestions. Moreover this strain gives an interesting possibility that it rapidly makes mosquitoes sick, reduces its blood feeding willingness and disrupting its reproduction propensity. These unique properties of *C. violaceum* combined with its environmentally friendly and logistically simple considerations could render this strain a highly potent malaria vector control biopesticide. It should be noted that further investigations are required in other to withdraw more conclusion on the efficacy of *C. violaceum* to control malaria vector. The strain of *C. violaceum* although identified to level of species the Vitek 2 compact automated system, it would be important to proceed to the sequencing of the genome of this bacterium and compare it with other species of *Chromobacterium sp*. This will certainly learn us on the specificity of this strain and the role of some of genes in its properties.

## Supplementary information

**Additional file 1.** The results of the identification of Chromobacterium violaceum strains isolated from mosquitoes using Vitek Software.

**Additional file 2.** The R_Codes of data Analysis.

## Data Availability

The supplementary R codes and data for all analyses in this article are in supplemental files and could also be available upon request to the corresponding authors.

## Abbreviations

*C. violaceum*: *Chromobacterium violaceum*
LT_50_: Is the median (50%) Lethal Time (time until death) after exposure of a mosquito to bacterial infections.
LT_80_: Is the 80% Lethal Time (time until death) after exposure of a mosquito bacterial infections
