# Supplementary material for: Infection of highly insecticide-resistant malaria vector Anopheles coluzzii with entomopathogenic bacteria Chromobacterium violaceum reduces its survival, blood feeding propensity and fecundity
Source: Malar J. 2020 Oct 2;19:352. doi: 10.1186/s12936-020-03420-4 (PMC7530970; doi:10.1186/s12936-020-03420-4)
Supplement: Supplementary file 1 — Additional file 1. The results of the identification of Chromobacterium violaceum strains isolated from mosquitoes using Vitek Software. [file 12936_2020_3420_MOESM1_ESM.pdf]

CENTRE MURAZ

Rapport du laboratoire

N° Client bioMérieux :
Référence du système :

Imprimé 8 avr. 2017 08:14 CDT
Imprimé par : arthur
Version du rapport : 1 / 1
ID du patient :

Nom du patient :
Groupe d'isolats : 1-1

Type de carte : GN Instrument de test : 0000170876CD (14582)

Profil biochimique : 0063000150440010
Numération :

**Commentaires :**	

Informations sur l'identification	**Carte :**	GN	**N° de lot :**	241388840	**Péremption :**	6 août 2017 13:00 CDT
	**Terminée le :**	7 avr. 2017 21:06 CDT	**État :**	Final	**Heure de l'analyse :**	5,00 heures
Germe sélectionné	98% de probabilité		**Chromobacterium violaceum**			
	**Profil biochimique :**	0063000150440010			**Fiabilité :**	Excellente identification
**Germe SRF**						
**Germes identifiés et tests discriminants :**						
**Commentaire sur l'ident. :**						
**Tests à l'encontre**						

Détails biochimiques																	
2	APPA	-	3	ADO	-	4	PyrA	-	5	IARL	-	7	dCEL	-	9	BGAL	-
10	H2S	-	11	BNAG	+	12	AGLTp	+	13	dGLU	+	14	GGT	+	15	OFF	-
17	BGLU	-	18	dMAL	-	19	dMAN	-	20	dMNE	-	21	BXYL	-	22	BAlap	-
23	ProA	-	26	LIP	-	27	PLE	-	29	TyrA	+	31	URE	-	32	dSOR	-
33	SAC	+	34	dTAG	-	35	dTRE	+	36	CIT	-	37	MNT	-	39	5KG	-
40	ILATk	-	41	AGLU	-	42	SUCT	+	43	NAGA	-	44	AGAL	-	45	PHOS	+
46	GlyA	-	47	ODC	-	48	LDC	-	53	IHISa	-	56	CMT	-	57	BGUR	-
58	O129R	+	59	GGAA	-	61	IMLTa	-	62	ELLM	-	64	ILATa	-			

Version de VITEK 2 Systems installée : 07.01
Norme d'interprétation des CMI :
Nom du jeu de paramètres AES :

Politique d'interprétation thérapeutique :
Dernière modification du paramètre AES :

Supplementary file 1: Result of the identification of *Chromobacterium violaceum* strains isolated from mosquitoes using Vitek Software.

The efficiency of the VITEK® 2 COMPACT instrument and VITEK® 2 PC software offers reliable bacterial identification (ID) and antibiotic susceptibility testing (AST). The instrument also lets us enhance laboratory efficiencies with reduced hands-on time and rapid reporting capabilities. Here, the result sheet contains information on bacterial species identification and the biochimical details of identifed bacteria

Supplementary file 2: R_Codes of data Analysis

Below are the R scripts that we wrote for data (Supplementary file 3) analysis.

```
### Data analysis Infection of malaria vector Anopheles
 coluzzii with an environment friendly entomopathogenic
 bacteria Chromobacterium violaceum reduces blood feeding
 propensity and fecundity ####
## Data Bacteria Analysis/ Mortality serial dilution
# Data importation
Mdat <- read.csv("~/Desktop/CSP_Serial_edit - copie.csv",
 sep=";")
View(Mdat)
# Data cleanning and processing to analysis
str(Mdat)
summary(Mdat)
colnames(Mdat)
colnames(Mdat)[2]="Replicate"
colnames(Mdat)
library(reshape2)
Mdat2=melt(Mdat,colnames(Mdat)[c(1,2)],colnames(Mdat)[c(3:7)])
View(Mdat2)
library(plyr)
df2=ddply(Mdat2, .(Replicate,variable), transform,
 nval=sum(value, na.rm=TRUE))
View(df2)
df3=ddply(df2, .(Replicate,variable), transform,
 Dead=cumsum(value))
View(df3)
df3$Survival=(1-df3$Dead/df3$nval)
View(df3)
colnames(df3)[3]="Treatments"
View(df3)
df4=subset(df3, Day!="Alives")
df4$Day=as.numeric(as.character(df4$Day))
View(df4)
df5=ddply(df4, .(Day,Treatments), summarize,
 mean=mean(Survival), replicates=length(Survival),
```

```
 se=sd(Survival)/sqrt(length(Survival)))
View(df5)
library(ggplot2)
library(scales)
limits=aes(ymax=mean+se, ymin=mean-se)
theme = theme_bw()+theme(text = element_text(size=25),
 axis.title.x = element_text(size=25), axis.title.y =
 element_text(size=25), title = element_text(size=25),
 legend.title = element_text(size=25), legend.text =
 element_text(size=20))
levels(df5$Treatments)
cbPalette <-
 c("#3498DB","#4A235A","#7D3C98","#A569BD","#E8DAEF")
Plt=ggplot(df5, aes(Day, mean,
 color=Treatments))+geom_line(size=2)+geom_errorbar(limits,
 width=.1,
 size=1)+theme+scale_colour_manual(values=cbPalette)+ylab("Mean
 Survival")+xlab("Days")+scale_y_continuous(labels=percent)
summary(Plt)
#Displays the plot
Plt
library(plotly)
ggplotly(Plt)
Plt
#Calculate LT80s
####Load packages####
library(tidyverse)
library(reshape2)
library(plyr)
library(scales)
library(survival)
library(MASS)
View(df4)
LTdat=df4
View(LTdat)
LTdat$Alive=LTdat$nval-LTdat$Dead
View(LTdat)
attach(LTdat)
surv.per=0.20
colnames(LTdat)
LTdat2=ddply(LTdat, .(Treatments, Replicate), summarize,

 LT=as.numeric(dose.p(glm(cbind(Alive,Dead)~Day,binomial),p=sur
 v.per)))
LTdat2[LTdat2$LT>14 | LTdat2$LT<0,]$LT=NA
LT80.Error=ddply(LTdat2, .(Treatments), summarize, "LT80
 Mean"=mean(LT,na.rm=T), se=sd(LT,
```

```
 na.rm=T)/sqrt(length(LT[!is.na(LT)])),
 Replicates=length(LT[!is.na(LT)]))
View(LT80.Error)
### Comparisons LT80
View(LTdat2)
CvLTdat2=subset(LTdat2,Treatments!="Control")
View(CvLTdat2)
pairwise.t.test(CvLTdat2$LT, CvLTdat2$Treatments,
 p.adj="none")
# Pairwise comparisons using t tests with pooled SD
# data:  CvLTdat2$LT and CvLTdat2$Treatments
#    C1     C2     C3
# C2 0.0444 -      -
# C3 0.0122 0.2200 -
# C4 0.0006 0.0023 0.0054
# P value adjustment method: none
### Code of Analysis Blood feeding###
# Data analysis tunnel test_chromobacterium
Tdat <- read.csv("~/Desktop/Blood_feeding_edit_Cv_2.csv",
 sep=";")
View(Tdat)
colnames(Tdat)
dat=Tdat[c(2,4:9)]
View(dat)
colnames(dat)
str(dat)
colnames(dat)[4]="Empty"
colnames(dat)[5]="Status"
colnames(dat)[6]="GP"
colnames(dat)[7]="Vitality"
colnames(dat)
levels(dat$Status)
levels(dat$Status)=c("Sugarfed", "Sugarfed", "Unfed", "Unfed")
levels(dat$Status)
levels(dat$Vitality)=c("Alive", "Dead", "Alive", "Dead")
View(dat)
colnames(dat)
library(reshape2)
dat=melt(dat, colnames(dat)[c(1:3, 5, 7)],
 colnames(dat)[c(4,6)])
View(dat)
library(plyr)
colnames(dat)[2]="DaysPostInfection"
colnames(dat)[3]="Treatment"
colnames(dat)[1]="Replicate"
View(dat)
colnames(dat)
```

```
dat1=ddply(subset(dat, Vitality=="Alive"), .(Replicate,
 DaysPostInfection, Treatment, variable), summarize,
 value=sum(value))
View(dat1)
dat2=ddply(dat1, .(Replicate, DaysPostInfection, Treatment),
 transform, Percent=value/sum(value))
View(dat2)
dat3=ddply(dat2, .(DaysPostInfection, Treatment, variable),
 summarize, Mosquitoes=mean(Percent),
 se=(sd(Percent)/sqrt(length(Percent))),
 Replicates=length(value))
View(dat3)
levels(dat3$Treatment)[1]="C.violaceum"
levels(dat3$Treatment)[2]="Control"
levels(dat3$Treatment)
# We can start the t.test
View(dat1)
subsetters=dat1$Treatment=="control"&dat1$variable=="GP"
testdat=dat1[subsetters,]
View(testdat)
pairwise.t.test(testdat$value,
 testdat$DaysPostInfection,p.adj="none")
# Below are results
# Pairwise comparisons using t tests with pooled SD
#data:  testdat$value and testdat$DaysPostInfection

#   3    4    5    6    7    8
#4  0.85 -    -    -    -    -
#5  0.96 0.82 -    -    -    -
#6  0.17 0.13 0.19 -    -    -
#7  0.93 0.78 0.96 0.20 -    -
#8  0.82 0.68 0.85 0.25 0.89 -
#9  0.93 0.78 0.96 0.20 1.00 0.89
# P value adjustment method: none
subsetters=dat1$Treatment=="CSP"&dat1$variable=="GP"
testdat2=dat1[subsetters,]
pairwise.t.test(testdat2$value,
 testdat2$DaysPostInfection,p.adj="none")
#Pairwise comparisons using t tests with pooled SD
#data:  testdat2$value and testdat2$DaysPostInfection
#   3       4       5       6       7       8
# 4 0.04402 -       -       -       -       -
# 5 0.07216 0.80567 -       -       -       -
# 6 0.00247 0.20920 0.13736 -       -       -
# 7 0.00018 0.02620 0.01528 0.28539 -       -
# 8 0.00018 0.02620 0.01528 0.28539 1.00000 -
# 9 7.9e-05 0.01225 0.00698 0.16321 0.73071 0.73071
```

```
# P value adjustment method: none
### Comparisons in GP between days post infections
subsetters=dat$DaysPostInfection==3 & dat$variable=="GP"
testdat=dat[subsetters,]
pairwise.t.test(testdat$value, testdat$Treatment)
# Results below
#airwise comparisons using t tests with pooled SD
# data:  testdat$value and testdat$Treatment
#         CSP
#control 0.91
#P value adjustment method: holm
subsetters=dat$DaysPostInfection==4 & dat$variable=="GP"
testdat=dat[subsetters,]
pairwise.t.test(testdat$value, testdat$Treatment)
#Results below
# Pairwise comparisons using t tests with pooled SD
# data:  testdat$value and testdat$Treatment
#       CSP
# control 0.45
# P value adjustment method: holm
subsetters=dat$DaysPostInfection==5 & dat$variable=="GP"
testdat=dat[subsetters,]
pairwise.t.test(testdat$value, testdat$Treatment)
# Pairwise comparisons using t tests with pooled SD
#data:  testdat$value and testdat$Treatment

#       CSP
#control 0.54
# P value adjustment method: holm
subsetters=dat$DaysPostInfection==6 & dat$variable=="GP"
testdat=dat[subsetters,]
pairwise.t.test(testdat$value, testdat$Treatment)
# Pairwise comparisons using t tests with pooled SD
# data:  testdat$value and testdat$Treatment
#        CSP
#control 0.42
#P value adjustment method: holm
subsetters=dat$DaysPostInfection==7 & dat$variable=="GP"
testdat=dat[subsetters,]
pairwise.t.test(testdat$value, testdat$Treatment)
# pairwise comparisons using t tests with pooled SD
# data:  testdat$value and testdat$Treatment
#         CSP
# control 0.18
# P value adjustment method: holm
subsetters=dat$DaysPostInfection==8 & dat$variable=="GP"
testdat=dat[subsetters,]
```

```
pairwise.t.test(testdat$value, testdat$Treatment)
# pairwise comparisons using t tests with pooled SD
# data:  testdat$value and testdat$Treatment
#       CSP
# control 0.2
# P value adjustment method: holm
subsetters=dat$DaysPostInfection==9 & dat$variable=="GP"
testdat=dat[subsetters,]
pairwise.t.test(testdat$value, testdat$Treatment)
# pairwise comparisons using t tests with pooled SD
# data:  testdat$value and testdat$Treatment
#        CSP
#control 0.12
#P value adjustment method: holm
##Plot according days post infection
library(ggplot2)
library(scales)
View(dat1)
levels(dat1$Treatment)[1]="C.violaceum"
plt=ggplot(dat1, aes(Treatment, value,
 fill=variable))+geom_bar(stat="identity",
 position="dodge")+facet_wrap(~DaysPostInfection)
plt
limits=aes(ymax=Mosquitoes+se, ymin=Mosquitoes-se)
theme = theme_bw()+theme(text =
 element_text(size=25),axis.title.x = element_text(size=30),
 title = element_text(size=35))
levels(dat3$Treatment)
cbPalette <- c("#7D3C98","#007acc")
plt=ggplot(subset(dat3, variable=="GP"),
 aes(DaysPostInfection, Mosquitoes,
 color=Treatment))+geom_line(size=3)+geom_errorbar(limits,
 width=.2,
 size=2)+theme+scale_colour_manual(values=cbPalette)+xlab("Days
 post bacterial infection")+ylab("Blood feeding
 interest")+scale_y_continuous(labels=percent)
plt
library(plotly)
ggplotly(plt)
### Modelisation Mortaltiy and blood feeding
#Combine host preference and mortality for transmission
View(df5)
sdf=subset(df5,Treatments!="C1")
View(sdf)
sdf1=subset(sdf,Treatments!="C3")
View(sdf1)
sdf2=subset(sdf1,Treatments!="C4")
```

```
View(sdf2)
Mort=rbind(subset(sdf2, Day==3), subset(sdf2, Day==4),
 subset(sdf2,Day==5), subset(sdf2, Day==6), subset(sdf2,
 Day==7))
View(Mort)
Mort=Mort[1:3]
View(Mort)
View(dat3)
levels(dat3$Treatment)[1]="C2"
Trans=dat3
levels(Trans$Treatment)[2]="Control"
View(Trans)
Trans$Day=as.numeric(Trans$DaysPostInfection)
Trans=subset(Trans, variable=="GP")
View(Trans)
Trans=Trans[c(2,4,7)]
View(Trans)
colnames(Trans)[2]="Interest"
View(Trans)
Trans1=subset(Trans,Day!=8)
Trans2=subset(Trans1,Day!=9)
View(Trans2)
View(Mort)
dat.m=merge(Mort, Trans2)
View(dat.m)
dat.m$Transmission=dat.m$mean*dat.m$Interest
View(dat.m)
levels(dat.m$Treatment)
#Plot transmission data
cbPalette <- c("#7D3C98","#3498DB")
theme = theme_bw()+theme(text = element_text(size=20),
 axis.title.x =
                           element_text(size=30), axis.text.x
 = element_text(angle=90, hjust=1,

 vjust=.5, size=25), axis.text.y = element_text(size=25), title
 =
                           element_text(size=35), legend.title
 = element_text(size=25), legend.text
                        = element_text(size=20))
t.plt2=ggplot(dat.m)+geom_area(aes(Day,mean,
 color=Treatments,fill=Treatments,
 position="identity"),alpha=0.4)+scale_fill_manual(values=cbPal
 ette)+geom_area(aes(Day, Transmission, color=Treatment,
 fill=Treatment,position="identity"),alpha=0.5)+geom_hline(yint
 ercept=.2, size=1,
 color="white",linetype="dashed")+geom_hline(yintercept=.5,
```

```
 size=1,
 color="white",linetype="dashed")+theme+scale_colour_manual(val
 ues=cbPalette)+xlab("Days after exposure")+ylab("Percent
 survival")+scale_y_continuous(breaks=pretty_breaks(n = 10),
 labels=percent)+scale_x_continuous(breaks=0:max(dat.m$Day))+fa
 cet_wrap(~Treatment)
t.plt2

View(InsectSprays)
boxplot(count ~ spray, data = InsectSprays, outpch = NA)
stripchart(count ~ spray, data = InsectSprays,
           vertical = TRUE, method = "jitter",
           pch = 21, col = "maroon", bg = "bisque",
           add = TRUE)
fcdat <- read.csv("~/Desktop/fcdat.csv", sep=";")
View(fcdat)
colnames(fcdat)
fcdat$Prop_eggs=fcdat$Nb.Eggs/fcdat$total.mosquito
View(fcdat)
colnames(fcdat)
fcdat$Vitality=(fcdat$Nb.Larvae/fcdat$Nb.Eggs)*100
View(fcdat)
boxplot(Prop_eggs ~ Treatment, data = fcdat, outpch = NA)
stripchart(Prop_eggs ~ Treatment, data = fcdat,
           vertical = TRUE, method = "jitter",
           pch = 21, col = "maroon", bg = "bisque",
           add = TRUE)
library(ggplot2)
fec <- read.csv("~/Desktop/fec.csv", sep=";")
View(fec)
colnames(fec)
fec$Vitality=fec$Nb.Larvae/fec$Nb.eggs
View(fec)
levels(fec$Treatment)[1]="C.violaceum"
colnames(fec)
library(ggplot2)
library(scales)
cbPalette <- c("#7D3C98","#3498DB")
theme = theme_bw()+theme(text = element_text(size=20),
 axis.title.x =
                          element_text(size=30), axis.text.x
 = element_text(angle=0, hjust=.5,

 vjust=.5, size=25), axis.text.y = element_text(size=25), title
 =
                          element_text(size=35), legend.title
 = element_text(size=25), legend.text
```

```
                              = element_text(size=20))
Plt<-
 ggplot(fec,aes(Treatment,Vitality,fill=Treatment))+geom_boxplo
 t()+theme+scale_colour_manual(values=cbPalette)+xlab("Treatmen
 t")+ylab("Percent
 Vitality")+scale_y_continuous(breaks=pretty_breaks(n = 10),
 labels=percent)
Plt+scale_fill_manual(values=c("#7D3C98","#3498DB"))
View(fec)
fec=fec[2:6]
summary(fec)
C.fec=subset(fec,Treatment=="C.violaceum")
View(C.fec)
summary(C.fec)
Ctrl.fec=subset(fec,Treatment=="Control")
View(Ctrl.fec)
summary(Ctrl.fec)
library(ggplot2)
library(scales)
cbPalette <- c("#7D3C98","#3498DB")
theme = theme_grey()+theme(text = element_text(size=20),
 axis.title.x =
                                element_text(size=30), axis.text.x
 = element_text(angle=0, hjust=.5,

 vjust=.5, size=25), axis.text.y = element_text(size=25), title
 =
                                element_text(size=35), legend.title
 = element_text(size=25), legend.text
                              = element_text(size=20))
Eplt=ggplot(fec, aes(x=Treatment, y=Nb.eggs,color=Treatment))
 +
  geom_boxplot(notch = TRUE)+

 geom_jitter(position=position_jitter(0.2))+theme+scale_colour_
 manual(values=cbPalette)+xlab("Treatment")+ylab("Number of
 eggs laid / Female")
Eplt
```
